# Population based case–control study of serious non-fatal motorcycle crashes

**DOI:** 10.1186/1471-2458-13-72

**Published:** 2013-01-25

**Authors:** Lesley Day, Michael G Lenné, Mark Symmons, Peter Hillard, Stuart Newstead, Trevor Allen, Rod McClure

**Affiliations:** 1 MUARC, Monash Injury Research Institute, Monash University, Melbourne, Australia; 2 School of Applied Media and Social Sciences, Monash University, Melbourne, Australia

**Keywords:** Motorcycle, Injury, Case–control, Road infrastructure, Speed

## Abstract

**Background:**

Motorcycle sales, registration and use are increasing in many countries. The epidemiological literature on risk factors for motorcycle injury is becoming outdated, due to changes in rider demography, licensing regulations, traffic mix and density, road environments, and motorcycle designs and technologies. Further, the potential contribution of road infrastructure and travel speed has not yet been examined.

**Methods/design:**

A population based case–control study together with a nested case-crossover study is planned. Cases will be motorcycle riders who are injured but not killed in a motorcycle crash on a public road within 150 km radius of Melbourne, Australia, and admitted to one of the study hospitals. Controls will be motorcycle riders who ride through the crash site on the same type of day (weekday or weekend) within an hour of the crash time. Data on rider, bike, and trip characteristics will be collected from the participants by questionnaire. Data on crash site characteristics will be collected in a structured site inspection, and travel speed for the cases will be estimated from these data. Travel speed for the controls will be measured prior to recruitment with a radar traffic detection device as they ride through the crash site. Control sites for the case-crossover study will be selected 1 km upstream from the crash site and matched on either intersection status or road curvature (either straight or cornered). If the initial site selected does not match the case site on these characteristics, then the closest matching site on the case route will be selected. Conditional multivariate logistic regression models will be used to compare risk between the matched case and control riders and to examine associations between road infrastructure and road environment characteristics and crash occurrence. Interactions between type of site and speed will be tested to determine if site type is an effect modifier of the relationship between speed and crash risk. The relationship between rider factors and travel speed generally will be assessed by multivariate regression methods.

**Discussion:**

In the context of the changing motorcycling environment, this study will provide evidence on contemporary risk factors for serious non-fatal motorcycle crashes.

## Background

Powered two-wheeler vehicles will be an integral part of the global transport future. Consistent with international experience, motorcycle sales, registrations and use are all increasing in Australia [1,2]. Practicality underpins some of this growth as motorcycles are a relatively cheap and attractive transport option for dealing with traffic congestion, uncertain fuel costs, limited inner-city parking, dissatisfaction with public transport, and environmental concerns. There is also, for some riders at least, a lifestyle element due to the challenges and enjoyment that can be derived from riding, and this is a likely factor in the growth in particular in older adults returning to riding or taking it up for the first time [2].

Motorcycle riders and pillion passengers are a high risk crash and injury group and are over-represented, compared with other road users, in both fatal and non-fatal injurious crashes, even when travel exposure is taken into account [1]. In Australia, for example, motorcycle fatality and serious injury rates per kilometres travelled are 29 and 37 times higher, respectively, than for other vehicle types [3,4]. Trends in motorcycle fatality rates per registered motorcycles have recently been declining, but as a consequence of increased exposure, and the dramatic improvements in motor car safety over recent decades, motorcycle fatalities are comprising an increasing proportion of road deaths [2].

Consistent with the ageing population, there has been a demographic shift in rider fatalities with increases of 34% and 24% respectively in the 60–69 and 70+ age groups between 2003 and 2008 [3]. These demographic and exposure trends, and the high fatality and injury risk associated with motorcycle riding, have prompted Australian road and transport authorities to prioritise motorcycle safety in current road safety strategies [5,6].

Several risk factors for motorcycle injury have been identified in epidemiological studies in Europe, Asia, Australia and New Zealand [7-16]. These were conducted more than 10 years ago and while some of the findings remain relevant, others are limited by recent changes in the demographic distribution of riders, licensing regulations, traffic mix and density, road environments, the emergence of new motorcycle designs and technologies and cultural differences. Importantly, the potential contribution of road infrastructure and travel speed was not examined in these previous studies.

Road infrastructure is a particularly important factor in crash causation and consequent injury severity [17]. The role of infrastructure in motorcycle crashes is not understood to the same level as it is for crashes involving other types of motor vehicle, but it is likely to be more important due to the inherent instability of a motorcycle and the relative lack of rider protection in the event of a crash.

Objectively measured speed and its interaction with other variables such as age, experience and prevailing conditions have not been adequately examined as a risk factor for motorcycle crashes. In particular, the roles of excessive (exceeding the posted speed limit) and inappropriate speed (inappropriate for the conditions and experience of the rider) in crash causation are current topics of interest to road safety agencies and the motorcycling community.

Our previous study in Victoria examined the role of inappropriate and excessive speed in motorcycle fatalities through a retrospective analysis of coronial and police-reported data [18]. Where speed involvement could be ascertained, around half of the crashes involved riders exceeding the speed limit, and 70% involved riding at speeds too fast for the conditions. Rider age, gender, licence status and engine capacity were associated with speed involvement in crashes. Given the focus on fatalities, data on driver behaviour (particularly human error and the pre-conditions) prior to the crash was incomplete – an important limitation as driver behaviour and human error are likely to be related to speed-involvement in a crash [19]. Further research utilising study designs which enable the inclusion of these important behavioural factors, and other environmental and infrastructure-related factors, and their co-occurrence with speed are warranted.

Taking a holistic systems view in the research is consistent with a key principle of the safe system framework - the inter-related nature of the components [20]. Road infrastructure, road layout and the environment around the road all play separate and interacting roles in determining road user behaviour, including travel speed selection. Reliable contemporary research evidence regarding motorcycle crash risk is required to guide appropriate policy, particularly in the areas of road environment and the setting and enforcement of speed limits, as well as informing education and training programs.

We are therefore undertaking a case–control study of motorcycle crashes which includes in-depth examination of the crash scene to estimate crash speed and interviews with the rider to reveal the circumstances surrounding the appropriateness of the speed at the time of the crash, to compare with a random sample of non-crashed motorcycle riders. The study will establish the contemporary evidence on rider risk factors relevant to the changing rider population and their environment, and quantify the association between travel speed and road infrastructure, and increased risk of a serious motorcycle crash.

## Design, methods and discussion

### Conceptual framework and research questions

The causation of on-road motorcycle crashes involves a complex interplay between the rider, environment (physical, social, legal), and motorcycle related factors. There are three main foci for this study: rider related factors, travel speed, and road infrastructure. The main research questions are:

1. What rider related factors are significantly associated with serious motorcycle related crashes and how do those factors interact?

2. What is the role of travel speed, both in excess of the posted speed limit and inappropriate for the conditions, in serious motorcycle crashes?

3. What is the relationship between rider related factors and travel speed in serious motorcycle crashes?

4. What road infrastructure and road environment factors are significantly associated with serious motorcycle related crashes, and how do these interact with travel speed?

### Design

This is a population based case control study together with a nested case-crossover study. The population based study is designed to answer research questions 1–3. The case-crossover study is designed to answer research question 4. The population base is motorcyclists riding on public roads between the hours of 0600 and 2400 within a 150 km radius of Melbourne (the study region), the second most populated city in Australia. A motorcycle is defined as a powered two wheel vehicle which is registrable for use on Victorian roads, including mopeds and scooters.

### Participants and recruitment

Cases are motorcycle riders from the population base who are injured in a motorcycle crash and admitted to one of the 10 major metropolitan Melbourne hospitals or five regional hospitals within the study area. These hospitals account for 78% of motorcycle crash admissions (unpublished data from the Victorian Injury Surveillance Unit). Potential cases will be identified from the hospital data systems and invited to participate by the study nurse, similar to previous studies [21]. The majority of potential cases will be approached while still in hospital, however, those who are missed will receive a letter giving them the opportunity to opt out of telephone follow-up regarding study participation. Fatally injured riders will not be included in the study. The relevant databases (such as the National Coronial Information System) do not hold sufficient reliable data on the required factors [18], and data obtained from next of kin are likely to be inaccurate or missing for important variables in our study.

Potential control participants are riders who pass through the case crash site on the same type of day (week-day, Saturday, Sunday) within an hour of the crash time. These riders will be photographed (time-stamped side and rear views) to record the registration number plate of the motorcycle. The state road authority, VicRoads, will then post an invitation package including the questionnaire to the registered owner, except in instances where the motorcycle is recorded as stolen. A tripod mounted camera will be set up as close to the crash site as possible. The camera is a professional digital single lens reflex (SLR) camera with an autofocus telephoto lens, (Nikon D3S, Nikkor 35-135 mm f3.5-4.5, Nikon Corporation, Tokyo, Japan). The camera has modifications to capture still images of moving vehicles under a wide range of light conditions, including darkness.

If a suitable safe location for control recruitment cannot be identified on the same road a suitable alternative will be used. For example, for a freeway with no emergency stopping lane, a recruitment site would be established on the nearest accessible road on the route taken by the crashed rider the next arterial.

Due to the lower response rates normally achieved with mailed questionnaires, compared with face to face recruitment, participating control riders will be eligible for a monthly lottery for a A$200 credit voucher.

### Data collection

#### Module 1: rider questionnaires

Information relating to the rider, motorcycle, trip factors, and, for cases only, the crash circumstances and outcomes, will be collected by structured questionnaires, based on previous research [13,15,22,23], administered either in person or by telephone by the study nurse for the case riders, and by mail or on-line for the control riders. Previous research indicates that comparable information in injury studies can be collected via these mixed modes [24].

Rider factors include: demographics; height and weight; medical conditions; reasons for riding; riding and driving experience; rider training; lifetime and recent exposure; infringement and crash history; exposure to other road user behaviour which puts rider at risk; rider behaviour (modified Motorbike Rider Behaviour Questionnaire [25]); thrill seeking and risk acceptance [26]. Injury details and alcohol and drug test results will be extracted from the medical record for the case riders.

Motorcycle factors include: motorcycle type (eg. sports, cruiser, scooter/moped), safety features; engine capacity; modifications; maintenance history; and rider familiarity. Control participants will be asked to provide the motorcycle registration number to allow matching with the recorded travel speed.

Trip factors include: weather and light conditions; length and purpose; traffic density; presence of other riders; route familiarity; recent sleep alcohol and drug use; use of personal protective clothing and equipment and visibility (use of lights, clothing colour). If carrying a pillion, gender, height, weight, motorcycle licence status, experience with the rider will be recorded. Cases will be asked to trace their trip route on a map for the purposes of identifying control sites.

Crash circumstances and outcomes include: date and time; sequence of events; type and geometry of crash; and nature of injuries sustained. The reference trip for the control riders will be the trip on which they were observed riding through the case crash site.

#### Modules 2 and 3: site observations

Information relating to the characteristics of the case (module 2) and control (module 3) sites, will be collected during a site inspection using a structured data collection tool, the methods for which will be based on established protocols [22,27,28]. Control sites for the case-crossover study, will be 1 kilometre upstream from the case crash site on the case rider’s route, and will be matched on either intersection status or road curvature (classified as either straight or cornered). If the site 1 kilometre upstream does not match the case site on these characteristics, then the closest site that matches on intersection status or road curvature will be selected.

Site characteristics to be observed are: road type; shoulder width and condition; intersection type; surface; camber; curvature; topography; line markings; traffic control devices and other infrastructure; posted and recommended speed limit if present; foliage and other environmental objects; and sight distance. Module 2 will include an additional component to collect the data required for estimation of speed at the time of the case crash: tyre skid profile markings and skid lengths; gouges left by other parts of the motorcycle; distance travelled between impacts; slide distances; debris trail; and damage to road side objects (such as posts, trees).

Automated notifications from ambulance (daily) and police data systems (at the time a crash is logged) will allow case site inspections to be undertaken as soon as possible after the crash.

#### Module 4: crashed motorcycle observations

Inspection of the crashed motorcycles will yield additional data for estimation of case crash speed: speed on speedometer if locked, tyre dimensions, motorcycle gear and engine RPM (if indicated by locked tachometer). Gear ratio and final drive ratio will be obtained from the manufacturer’s specifications.

#### Module 5: travel speed observations and measurements

Control motorcyclist travel speeds will be measured when they are identified as potential participants riding through the case crash site. Travel speed on approach to the crash site will be recorded with a radar traffic detection device (Sierzega SR4, Sierzega Elektronik GmbH, Thening, Austria) positioned on the roadside. This device records date and time, classifies vehicles by length using a laser, measures speed from 3 to 254 km/hr with +/− 3% accuracy, reads multiple traffic lanes, and estimates traffic density. The radar will be unobtrusively secured at the roadside approximately 100 m upstream from the crash site. Speed will automatically be measured as motorcycles pass the device, prior to photographing. The average, median and range of speeds recorded by all passing motorcyclists will allow assessment of potential response bias in this key variable for participating control riders.

The motorcycle registration number (provided on the returned questionnaire) will be used to search for the relevant photograph (taken during control rider recruitment) and by matching the date and time of the photograph with the date and time of the speed measurement and vehicle type recorded by the radar device, travel speed measurements will be matched with the appropriate control motorcyclist.

For case riders, travel speed just prior to the crash will be estimated using data collected in module 2 and established protocols [22,27]. This includes estimation of impact speed using vehicle deformation data and manufacturer specification (eg. change in wheelbase) and/or rider dynamics (eg. slide distance, tumble distance), as well as estimation of velocity change prior to impact, using evidence at the crash site (eg. road surface characteristics and coefficient of friction, length of any skid marks). Due to the variability in the type of data available on which to base these calculations, the technical officer will estimate the degree of confidence in the calculation as a percentage.

### Sample size estimation

Using a two-sided significance level of 0.05 and 90% power, a sample size of 575 cases with a 1:1 case:control ratio would allow detection of an odds ratio of 2.0 or more, indicating moderate to strong associations, for risk factors with a prevalence between 10 and 85% in the control population, assuming a correlation of 0.3 maximum between the cases and controls on the matching factor.

### Planned analysis

Rider, site and crash characteristics, and injury outcomes will first be profiled using conventional descriptive statistics. Continuous variables will be summarised by mean, mode, standard deviation, skewness and percentile distributions, while categorical variables will be summarised using percentages. To compare risk between the matched case and control riders (research question 1), and assess the role of travel speed (research question 2), conditional multivariate logistic regression models will be used. The primary measure will be adjusted odds ratios and their associated significance values and 95% confidence intervals for the rider risk factors. The primary exposure measure of average weekly hours ridden will be treated as a modelling covariate. It will be constructed as a fractional polynomial to identify if the relationship between crash risk and exposure is linear or whether there is evidence of a low mileage bias.

Travel speed will be treated in the first instance as a continuous variable. In addition, two travel speed categorical variables will be created: excessive, defined as in excess of the posted speed limit, and inappropriate for the conditions. Speed inappropriateness for the conditions will be determined by an expert panel that will review the fixed physical and variable site characteristics and determine for each site an appropriate speed, within 10 km/h, to safely negotiate the crash site, for both a highly experienced rider and a novice. Panel members will be blinded to the case or control status of each site. Case speeds will necessarily be an estimate based on physical evidence, rather than a direct measurement as for the control speeds. There is some potential for systematic measurement error in speed estimation for cases in low speed crashes, where there is likely to be less physical evidence. Sensitivity analysis will be used to examine the influence on the odds ratio of systematic changes to case speed values determined in low speed crashes. Analysis excluding those cases for which there is a low level of confidence in speed estimation will also allow an assessment of the influence of such measurement error. Similarly, sensitivity analysis will facilitate an examination of the influence of potential response bias which may arise from the higher speed cases not participating (either declining or being too severely injured).

Conditional multivariate logistic regression models will also be used to examine associations between road infrastructure and road environment characteristics and crash occurrence (research question 4). Interactions between type of site and speed will be tested in the analysis models to determine if site type is an effect modifier of the relationship between speed and crash risk.

The relationship between rider factors and travel speed generally (research question 3) will be assessed by multivariate regression methods. Four models will be developed to analyse the relationship between rider factors and (1) mean travel speed (2) likelihood of exceeding the speed limit (3) likelihood of exceeding the speed limit by 25 km or more and (4) likelihood of travelling at an inappropriate speed. Logistic regression will be used for all except the first model for which linear regression will be utilised. Interaction between the predictive variables and case or control status will be examined in all four models. Known or suspected confounding factors will be included in the multivariate models to adjust for confounding if they alter the β co-efficient by more than 10% [29].

### Consultation and approvals

Key motorcyclist groups were consulted during the development of the research design, and two workshops were held with individual riders to seek input on the case and control questionnaires, and site and motorcycle inspection data items. The study has been reviewed and approved by the Monash University Human Research Ethics Committee (CF11/0989 – 2011000482), and the 15 hospital ethics committees. The need to streamline ethics applications for multicentre research, noted previously, remains [21]. The process of preparing the same application on different forms and with different standards for participant explanatory statements is inefficient, unnecessarily increases research costs, and introduces delays in the establishment phase.

## Conclusion

In the context of the changing motorcycling environment, this study will provide evidence on contemporary risk factors for serious non-fatal motorcycle crashes. It will examine two key aspects of motorcycle safety which have been previously incompletely investigated and which are current topics of debate: speed and road infrastructure. Motorcycle rider groups maintain that experienced motorcyclists are capable of handling higher speeds than the posted speed limit, and may justify exceeding the speed limit for this reason [30]. Road traffic and policing authorities maintain that beyond being a legal requirement, the speed limits are set at appropriate levels for safe travel. Robust evidence based results on the role of speed in motorcycle crashes, and the interaction of speed with factors such as age, experience and training, and motorcycle type will provide credible information to both riders and the authorities.

Motorcycle rider groups have also suggested that road infrastructure designed to mainly decrease motor vehicle crash risk, or occupant injury severity, is sometimes inappropriate for motorcyclists and may in fact increase their crash risk or injury severity. The proposed study is innovative in addressing the role of speed, identifying road infrastructure characteristics that pose excess risk for motorcyclists, and examining these factors within the safe system framework. Our methods for quantifying and determining the role of speed, and for assessing if speed was inappropriate for the conditions, will be the most rigorous and comprehensive applied to date. With the current policy emphasis on the safe system approach to road safety, results from this study will assist road safety authorities in optimising the management of the road system for safe outcomes for motorcyclists as well as other road users.

## Competing interests

The authors declare that they have no competing interests.

## Authors’ contributions

ML led the work from which the original study concept arose. LD and RM conceived the overall study design and contributed to development of data collection instruments. LD is the study chief investigator, is supervising the conduct of the study, and drafted the manuscript. ML selected the measures for fatigue and distraction. MS, RM and ML selected the measures for rider behaviour, thrill seeking and risk acceptance. SN selected exposure measures, devised the analysis plan and is supervising data collection, quality and analysis. PH selected the measures for site and motorcycle observations, and contributed to the methods for speed estimation. MS undertook a feasibility study on the methods for control travel speed observation. TA developed the detailed study protocol and instruments and is responsible for study execution. All authors read, provided critical appraisal and approved the final manuscript.

## Pre-publication history

The pre-publication history for this paper can be accessed here:

http://www.biomedcentral.com/1471-2458/13/72/prepub

## References

[B1] Peden M Scurfield R Sleet D Mohan D Hyder AA Jarawan E Mathers C World report on road traffic injury prevention 2004 World Health Organisation, Geneva

[B2] Haworth N Powered two wheelers in a changing world- challenges and opportunities Accid Anal Prev 2012 44 12 18 10.1016/j.aap.2010.10.031 22062331

[B3] Department of Infrastructure, Transport, Regional Development and Local Government Road deaths Australia 2008 statistical summary. Road safety report No. 4 2009 The Department, Canberra

[B4] Henley G Harrison JE Serious injury due to land transport accidents, Australia, 2006–07 2009 Australian Institute of Health and Welfare, Canberra

[B5] Victorian Government Victoria’s road safety and transport strategic action plan for powered two wheelers 2009–2013 2009 Victorian Government, Melbourne

[B6] Department of Transport and Main Roads Queensland motorcycle safety strategy 2009–2012 2009 Queensland Government, Brisbane

[B7] Lin M-R Chang S-H Pai L Keyl PM A longitudinal study of risk factors for motorcycle crashes among junior college students in Taiwan Accid Anal Prev 2003 35 243 252 10.1016/S0001-4575(02)00002-7 12504145

[B8] Zambon F Hasselberg M Socioeconomic difference and motorcycle injuries: age at risk and injury severity among young drivers. A Swedish nationwide cohort study Accid Anal Prev 2006 38 6 1183 9 10.1016/j.aap.2006.05.005 16806026

[B9] Association des Constructeurs Européens de Motocycles. MAIDS In-depth investigations of accidents involving powered two wheelers 2004 The Association, Brussels

[B10] Mullin B Jackson R Langley J Norton R Increasing age and experience: are both protective against motorcycle injury? a case–control study Inj Prev 2000 6 32 35 10.1136/ip.6.1.32 10728539 PMC1730576

[B11] Langley J Mullin B Jackson R Norton R Motorcycle engine size and risk of moderate to fatal injury from a motorcycle crash Accid Anal Prev 2000 32 659 663 10.1016/S0001-4575(99)00101-3 10908138

[B12] Wells S Mullin B Norton R Langley J Connor J Lay-Yee R Jackson R Motorcycle rider conspicuity and crash related injury: case–control study Br Med J 2004 37984.574757 EE 10.1136/bmj.37984.574757.EE PMC387473 14742349

[B13] Haworth N Smith R Brumen I Pronk N Case control study of motorcycle crashes 1997 The Federal Office of Road Safety, Canberra, Australia CR174

[B14] Haworth N Mulvihill C Crashes of older Australian riders. In proceedings of international motorcycle safety conference The human element: 28-30 march 2006; long beach, California 2006 The Motorcycle Safety Foundation, California http://www.msf-usa.org/imsc/index.html

[B15] Haworth N Mulvihill C Symmons M Motorcycling after 30 2002 Monash University Accident Research Centre, Melbourne Report No. 192

[B16] Sexton B Baughan C Elliot M Maycock G The accident risk of motorcyclists 2004 Summary of TRL Report TRL607, Berkshire UK

[B17] Vlahogianni E Yannis G Golias J Overview of critical risk factors in power-two-wheeler safety Accid Anal Prev 2012 49 12 22 22579296 10.1016/j.aap.2012.04.009

[B18] Stephan K Symmons M Hillard P Bohensky M Muir C Lenne MG Characteristics of fatal motorcycle crashes involving excessive and/or inappropriate speed 2008 Monash University Accident Research Centre, Melbourne

[B19] Phan V Regan M Leden L Mattson M Minton R Chattington M Basacik D Pittman M Baldanzini N Vlahogianni E Yannis G Golias J Rider/driver behaviours and road safety for PTW. Deliverable 1 of EU project 2-be-safe 2011 http://www.transport-research.info/upload/documents/201203/20120330_120438_76514_2BES-WP1.1_D1.pdf

[B20] Tingvall C Holst Von H, Nygren A, Thord R The zero vision—a road transport system free from serious health losses Transportation, traffic safety and health 1997 Springer–Verlag, Berlin 37 57

[B21] Day L Langley J Stathakis V Wolfe R Sim M Voaklander D Ozanne-Smith J Challenges of recruiting farm injury study participants through hospital emergency departments Inj Prev 2007 13 88 92 10.1136/ip.2006.013110 17446247 PMC2610597

[B22] Fildes B Logan D Hillard P Schofield P The Enhanced Crash Investigation Study (ECIS). Second European Transport Research Arena: 22nd April, 2008; Ljubljana, Slovenia 2008 European Commission, Brussels http://2008.traconference.eu/Programme/TRA2008PAPERS/tabid/159/Default.aspx

[B23] Symmons MA Mulvihill CM Haworth N Motorcycle crash involvement as a function of self assessed riding style and rider attitudes. Proceedings of the 2007 Australasian road safety research, policing and education conference: 17-19 October 2007, Melbourne http://arsrpe.acrs.org.au/pdf/RS07040.pdf]

[B24] Yorkston E Turner C Schluter P McClure RJ Validity and reliability of responses to a self-report home safety survey designed for use in a community-based child injury prevention programme Int J Inj Contr Saf Promot 2005 12 193 196 10.1080/1566097042000265791 16335438

[B25] Elliot MA Braughan CJ Sexton BF Errors and violation in relation to motorcyclists’ crash risk Accid Anal Prev 2007 39 491 499 10.1016/j.aap.2006.08.012 17034747

[B26] Turner C McClure R Quantifying the role of risk taking in the causation of serious road crash-related injury Accident Analysis and Prevention 2004 36 383 389 10.1016/S0001-4575(03)00031-9 15003583

[B27] Bellion P Project Y.A.M. (Yaw analysis methodology) vehicle testing and findings - Victoria police, accident investigation section, SAE technical paper 970955 1997 10.4271/970955

[B28] Hillard PJ Logan D Fildes B Dorn L The application of systems engineering techniques to the modelling of crash causation Driver behaviour and training: volume II 2005 Ashgate Publishing Ltd, Aldershot 407 416

[B29] Maldonado G Greenland S Simulation of study confounder selection strategies Am J Epidemiol 1993 123 11 923 936 8256780 10.1093/oxfordjournals.aje.a116813

[B30] Natalier K Motorcyclists’ interpretations of risk and hazard J Sociol 2001 37 65 80 10.1177/144078301128756201

